# Intra-tendinous Patellar Ganglion Cyst Maybe the Unusual Cause of Knee Pain: A Case Report

**DOI:** 10.7759/cureus.5467

**Published:** 2019-08-23

**Authors:** Mantu Jain, Nabin K Sahu, Sudarsan Behera, Rajesh Rana, Saroj K Patra

**Affiliations:** 1 Orthopaedics, All India Institute of Medical Sciences, Bhubaneswar, IND; 2 Trauma & Orthopaedics, All India Institute of Medical Sciences, Bhubaneswar, IND

**Keywords:** ganglion cyst, patellar intra-tendinous sheath, knee pain

## Abstract

Cystic lesion around knee usually presents as painless swelling and diagnosed incidentally by imaging for any internal derangement of the knee. Few cases presented with pain. Intra-tendinous patellar ganglion is very rare in location for the disease. Ganglionic cyst usually treated by aspiration followed by steroid and surgical excision in some cases. We reported a case with anterior knee pain due to patellar intra-tendinous ganglion cyst which treated conservatively with no recurrence even after one year.

## Introduction

A ganglion is a benign cystic mass containing clear high-viscosity mucinous fluid with a dense fibrous connective tissue capsule lined by flat spindle-shaped cells that is rich in hyaluronic acid and other mucopolysaccharides [1, 2]. Ganglia usually arises from periarticular locations with a variable for predilection intra-articular, extra-articular soft tissue, intraosseous and rarely from periosteal locations [1,3,4]. Majority of the patients are asymptomatic and diagnosed incidentally on imaging. Clinical presentation depends on the location and size of the ganglion. The dimensions of a ganglion may change over time, with increased size leading to increased symptoms which include pain, swelling and rarely peroneal nerve palsy [3,5]. On magnetic resonance imaging (MRI), ganglia appear as well-delineated, homogeneous, rounded or lobulated fluid collections. Peripheral fluid-filled pseudopodia and sharply defined internal septations are characteristic features of ganglia, which result in a “bunch of grapes” appearance [3]. Symptomatic ganglions are usually treated with intralesional aspiration with or without steroid injection or surgical excision. We reported a case of symptomatic patellar tendon ganglion which is a rare location and treated conservatively and symptomless even after one-year follow-up.

## Case presentation

**Figure 1:**
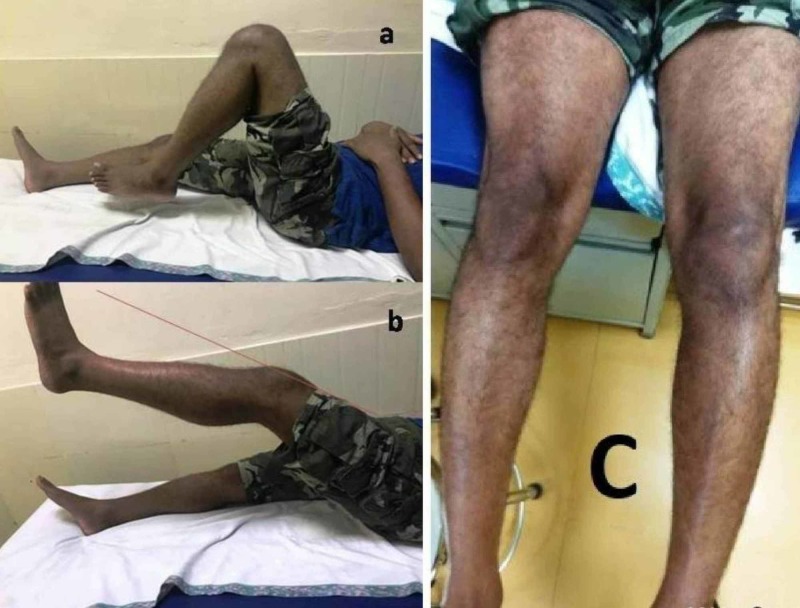
Clinical picture showing a) range of motion, b) extensor lag, and c) muscle wasting.

A 39-year-old boy presented to us with complaints of dull anterior knee pain for three months. He had visited several general practitioners for which he was given symptomatic analgesics. On examination, there was minimal swelling in form of the fullness of paratendon area and slight extensor lag with quadriceps muscle wasting (1 cm) without any instability with active range of motion 150-1200 (Figure 1).

**Figure 2:**
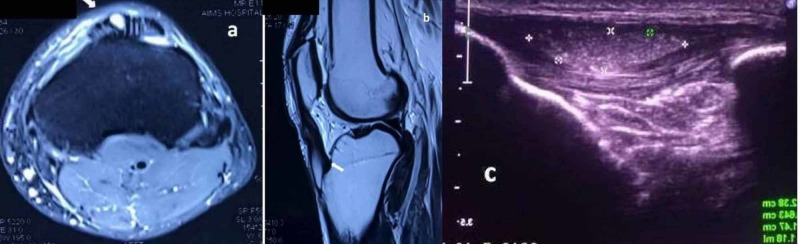
Imaging study a) Axial proton density fat saturated (PDFS) MRI; b) T2 sagittal image of the MRI shows a well-defined cystic lesion (arrows) within the patellar tendon (arrow head); c) ultrasonography confirms a moving well-defined echogenic lesion within the patellar tendon with approximate 1.2 ml volume.

An X-ray of the knee was unremarkable. MRI was advised and it showed a large intratendinous cyst of size 28 x 7 x 5.5 mm with high signal intensity on T2- and PD-WI occupied in the mid half of patella tendon and gradually thinning as terminating in the bony attachment (Figure 2).

An ultrasonography (USG) confirmed a moving well-defined echogenic lesion within the patellar tendon with approximate 1.2 ml volume. The radiological diagnosis was an intratendinous patellar ganglion (ITPG) cyst. The patient was explained regarding treatment option like aspiration and anti-inflammatory injections/surgery if conservative therapy fails, but he chooses to observe and follow up. Even after one-year follow-up the patient is doing well with no recurrence of symptom.

## Discussion

Ganglia arise from the mucoid degeneration and occurs in tendon sheath though they can occur in bones, joints and even other soft tissues [6]. Histologically, progressive liquefaction leads to splitting of the collagen fibers with the accumulation of this mucinous material into large vacuoles [7]. However, an intratendinous location is a rare finding. In the knee, infrapatellar fat pad, the alar folds, and the anterior cruciate ligament are recognized to degenerate into ganglion [8]. There are few case reports where the patellar tendon is described. Touraine et al. described a case of ITPG due to the patellar tendon-femoral condyle friction syndrome [9]. Mebis et al. described a similar entity with associated Osgood-Shatter disease [10]. However, the characteristic finding in this is lack of vascularization that can be seen on an ultrasound or contrast-enhanced ultrasound [11]. MRI can also help in delineating focal edema (in friction syndrome) or associated bony damage [12,13]. There is a lack of an algorithm for treatment due to rarity. Jose et al. reported that the evacuation with the injection of an anti-inflammatory agent may significantly improve clinical symptoms [14]. Mebis et al. had a surgical excision in their patient [10]. Our patient had mild extensor lag and even though we explained all treatment strategies, he chooses to manage with observation. We reserve surgery for the increase in size or functional disability considering the delicate area of presentation and the risks involved. The purpose is to highlight and sensitize the readers of this rare location of the commonly reported disease.

## Conclusions

The intra-tendinous patellar ganglion is a rare location for the disease but should be kept as one of the differential diagnosis for anterior knee pain. Imaging study is key to diagnosis in an unusual location.
